# Ribosomal protein L32 contributes to the growth, antibiotic resistance and virulence of *Glaesserella parasuis*

**DOI:** 10.3389/fvets.2024.1361023

**Published:** 2024-08-26

**Authors:** Qiaodan Chen, Bin Yu, Fei Su, Shiyi Ye, Lihua Xu, Xiufang Yuan, Shumin Wu, Hui Zhang, Junxing Li

**Affiliations:** ^1^College of Life Science and Engineering, Foshan University, Foshan, China; ^2^Institute of Animal Husbandry and Veterinary Medicine, Zhejiang Academy of Agricultural Sciences, Hangzhou, China

**Keywords:** ribosomal protein L32, *Glaesserella parasuis*, antibiotic resistance, growth rate, stress resistance

## Abstract

*Glaesserella parasuis* is the pathogen that causes Glässer’s disease in pigs, which is characterized by fibrinous polyserositis, arthritis and meningitis. Research on ribosomal protein L32 in microorganisms has mainly focused on regulating gene transcription and translation, but its effect on bacterial virulence is unclear. The role of *L32* gene in *G. parasuis* is not clear, and in order to study the function of *L32* gene, a suicide plasmid-mediated natural transformation method was used to construct a *L32* gene deletion mutant. We found that although *L32* was shown to be non-essential for cell proliferation, the growth curve of Δ*L32* is clearly different compared with that of ZJ1208. Δ*L32* produced more outer membrane vesicles (OMVs) with a variety of irregular shapes, but produced similar biofilm to the parental strain. Δ*L32* is more sensitive to osmotic pressure, oxidation pressure and heat shock stress. Meanwhile, Δ*L32* is significantly more susceptible to antimicrobials such as spectinomycin, apramycin, sulfafurazole, but not to other antibiotics used in this study. In the mouse challenge experiment, the mortality of mice infected with the mutant strain decreased by 40% compared to those infected with the wild-type strain, indicating that *L32* is a virulence-associated factor which contributes to bacterial fitness in host environments. The above results show that *L32* is important for the growth, stress resistance and virulence of *G. parasuis*, and this study also confirms for the first time that *L32* plays an important role in antibiotic resistance against aminoglycosides and sulfonamides.

## 1 Introduction

*Glaesserella parasuis* (*G. parasuis*), also formerly known as *Haemophilus parasuis*, is the pathogen of Glässer’s disease, which is characterized by fibrous polyserositis, arthritis and meningitis (1). *G. parasuis*, a Gram-negative small bacilli, belongs to the Pasteurella family and its growth is strictly depend on factor V (nicotinamide adenine dinucleotide, NAD) (2). It can be a commensal bacterium colonized in the upper respiratory tract of clinically normal pigs, or a pathogen causing severe systemic infection. The disease occurs in all the pork producing countries, and has become the most common bacterial disease in pig production and an important factor in the death of piglets during the nursery period (3). Due to the numerous serotypes of this bacterium, the cross-protection between serotypes is not ideal, resulting in a decrease in the effectiveness of inactivated vaccines for prevention and control of this disease (4). Up to now, the pathogenic mechanism of *G. parasuis* is not completely clear, which brings great challenges to the research of highly effective vaccines. At present, it is mainly controlled by antibiotics, which may lead to antibiotic abuse. Therefore, the pathogenesis, prevention and control of the disease are still difficult problems that need to be overcome.

Ribosome proteins play an important role in stabilizing rRNA structure to promote protein synthesis in ribosomes (5). *L32* is involved in the processing of rRNA precursors and the cleavage and translation of its own mRNA, and amino acid mutations in this protein lead to impaired growth rates (6, 7). In the study of the Gram-positive bacterium *Bacillus subtilis*, the *L32* gene deletion strain was still alive and had a similar growth rate to the wild strain (8). The *L32* protein has been shown to contribute to the SOL (solithromycin) resistance phenotype of *Streptococcus pneumoniae* (9). Macrolides have previously been shown to interact with many proteins in the 70S ribosome, including L32 (10). When screening nitrosoguanidine mutagenic *Escherichia coli* mutants, multiple ribosomal protein mutants, including L32, were screened by temperature-sensitive methods, indicating that L32 may affect the sensitivity of the bacterium to temperature (11).

The role of *L32* in *G. parasuis* is not clear. In this study, *L32* gene deletion mutant was constructed from a serovar 13 strain ZJ1208, and the role of *L32* gene in the growth, stress resistance, antibiotic resistance and pathogenicity of *G. parasuis* was explored.

## 2 Materials and methods

### 2.1 Bacterial strains, plasmids, and growth conditions

*Glaesserella parasuis* was cultured in Tryptone Soy Agar (TSA) or Tryptic Soy Broth (TSB) supplemented with 10 μg/mL NAD (Sangon Biotech) and 3% bovine serum at 37°C, and *E. coli* DH5α was cultured in Luria-Bertani broth (LB) at 37°C. A final of 30 μg/mL of kanamycin was added to the medium as needed. ZJ1208 is a serovar 13 *G. parasuis* clinical isolate with high natural transformation competency (12). The cloning vector is pEASY (Beijing TransGen Biotech), and pET-28a is a plasmid stored in the laboratory.

### 2.2 Construction of *L32* deletion mutant

**Figure 1:**
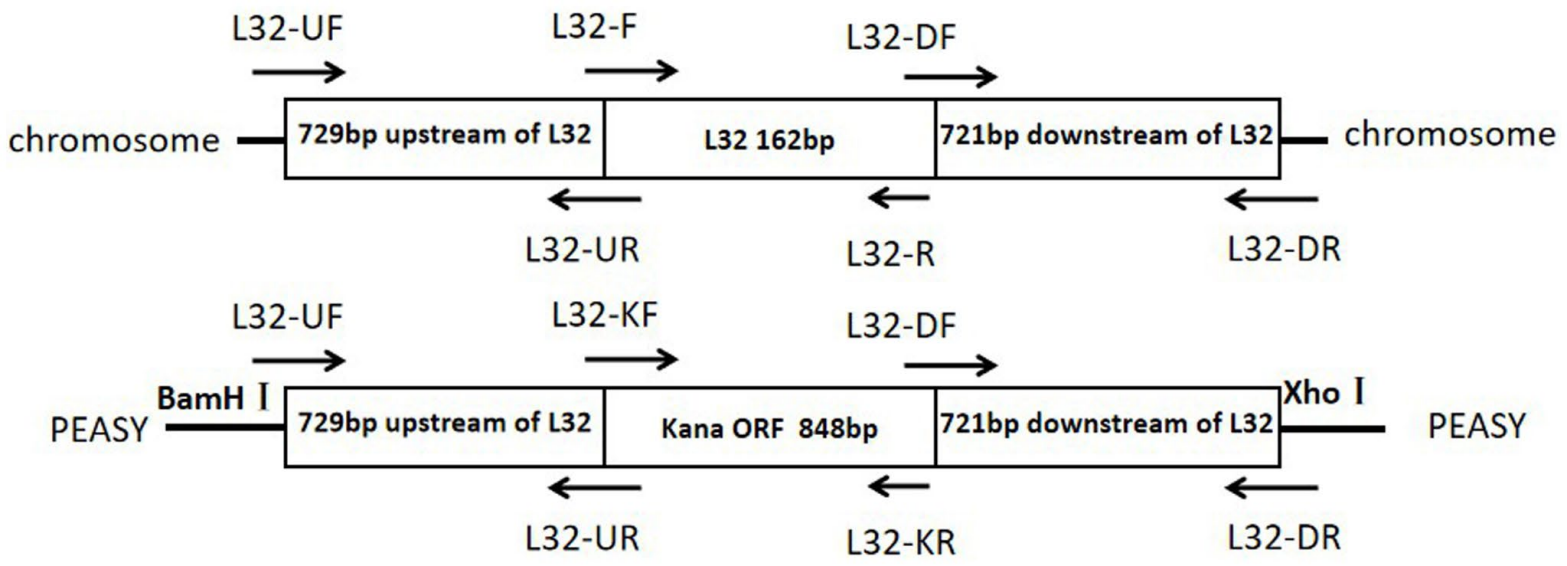
Schematic diagrams of the target region of the *Glaesserella parasuis* chromosome and of the pEASY recombinant plasmid.

The primers used in this study are listed in Table 1 and synthesized by Sangon Biotech. Using the genomic DNA of ZJ1208 strain as template, homologous arm fragments of upstream (729 bp) and downstream (721 bp) were amplified. Using pET-28a as template, the 848 bp KanR cassette was amplified with *L32*-KF and *L32*-KR primers. The three fragments were ligated by overlapping PCR with primers *L32*-UF and *L32*-DR, and the PCR products were ligated with cloning vector pEASY to obtain the recombinant plasmid pEASY-*L32* (Figure 1), the restriction sites *BamH* I and *Xho* I on the vector were used for double enzyme digestion. The recombinant plasmid was further confirmed by gene sequencing.

**Table 1:** Primers used in this study.

Primers	Sequence (5′ ~ 3′)	Fragment
*L32*-UF	ACGGCGAACGTTTTAACATATAAG	Upstream of *L32*729 bp
*L32*-UR	TGGCTCATTGGCTATACTCCTACTAGATCTAGTT	
*L32*-KF	GGAGTATAGCCAATGAGCCATATTCAACGGGAAA	KanR cassette 848 bp
*L32*-KR	CCTCTACGAGATCCAATTGATTAGAAAAACTCATCGAGCATCAAA	
*L32*-DF	TCAATTGGATCTCGTAGAGGTTTAAATTGACT	Downstream of *L32* 721 bp
*L32*-DR	GCAAGATGATTCATTAATTTATCCCC	
*L32*-F	ATGGCTGTTCAACAAAATAAG	*L32* 162 bp
*L32*-R	TTATTTGATTTTACGACCACGG	

The recombinant plasmid was transformed into *G. parasuis* using the natural transformation method as described (12). Briefly, 100 ng of plasmid was added to 300 μL of starvation medium induced competent *G. parasuis*, and the mixture was incubated at 37°C for 30 min. Then 200 μL of 80% glycerol solution was added and incubated at 25°C for 10 min, following by incubation at 37°C for 100 min in a horizontal shaker at 200 rpm. Finally, the cell culture was spread on TSA (supplemented with 30 μg/mL of kanamycin) and incubated at 37°C for 48 h. The primers listed above were then used to identify the insertion of KanR cassette and the deletion of *L32* gene.

### 2.3 RT-qPCR

To check the possible polar effect of L32 deletion, the gene expression level of the upstream gene *YceD* and the downstream gene *plsX* of *L32* gene were determined by real-time fluorescence quantitative PCR (RT-qPCR). The 16 s rRNA gene was selected as a reference housekeeping gene in PCR amplification. The primers, as shown in Table 2, were synthesized by Sangon Biotech.

**Table 2:** RT-qPCR primers used in this study.

Gene	Primer	Sequence (5′ ~ 3′)	Product length	Source
YceD	YceD-F	CTGTTGGTCAAATGCTGGGC	198 bp	This study
	YceD-R	GCAAATTGTCCGCCTGATCC		
plsX	plsX-F	CCAAACATTGATCGTCCCGC	137 bp	This study
	plsX-R	AACACATTGCCCATTTCCGC		
Housekeeping gene	16s-F	CGGGAAACTGTCGCTAAT	160 bp	Zhang et al. (13)
	16s-R	TGTGGCTGGTCATCCTCT		

Δ*L32* and ZJ1208 were cultured overnight in TSB medium supplemented with NAD and serum until the optical density at 600 nm reached approximately 0.7, the medium was removed, washed twice with PBS, and RNA was extracted using a combination of plasmid small amount extraction kit (Beijing Solarbio Science & Technology Co., Ltd.) and RNA extraction kit (Takara Bio Inc.). Finally, RNA was collected using 50 μL of eluent. The concentration of extracted RNA was measured using NanoVue Plus spectrophotometer (GE Healthcare, United States). Then store it at −70°C. The extracted RNA also needs to undergo gDNA removal. Finally, 900 ng of RNA is reverse transcripted into cDNA before undergoing qPCR.

### 2.4 Growth

Δ*L32* and ZJ1208 were cultured overnight in TSB medium supplemented with NAD and serum until the optical density at 600 nm reached approximately 0.5. Subsequently, they were inoculated into fresh TSB medium at a ratio of 1:50 and incubated at 37°C. MicroScreen high-throughput real-time microbial growth analysis system (MicroScreen HT, Gering) was employed to measure the OD_600_ every hour throughout the incubation period. This assay was repeated three times, and subsequently, a growth curve was constructed using the incubation time (h) as the *x*-axis and the average OD_600_ values as the *y*-axis.

### 2.5 Colony morphology

ZJ1208 and Δ*L32* were inoculated on TSA plates, and incubated at 37°C for 48 h, and colony morphology was observed under Chemiluminescence imaging system (Bio-Rad ChemiDoc XRS+).

### 2.6 Transmission electron microscope

The colonies of solid medium TSA and the logarithmic phase bacteria of liquid medium TSB were diluted with deionized water, and subsequently deposited onto polyvinyl formal-carbon-coated grids. Following natural drying, a brief 8-s staining with 2% phosphotungstic acid enabled direct observation of the bacteria under transmission electron microscopy (H-7650, Hitachi).

### 2.7 Osmotic pressure, oxidation tolerance and heat shock assays

Stress resistance assays were referred to the method described previously with slight modification (11, 14). *G. parasuis* ZJ1208 and Δ*L32* were cultured overnight, and the OD_600_ was adjusted to 0.65.

For the osmotic tolerance assay, 1 mL bacteria are resuspended in equal volume of TSB containing different concentrations of KCl (1, 2, and 3 M) and incubated at 37°C for 2 h.

For the oxidative stress assay, 1 mL bacteria were resuspended in equal volume of TSB containing different concentrations (10, 50, and 150 mM) H_2_O_2_ and incubated at 37°C for 1 h.

For heat shock assay, the bacteria culture was incubated in water bath at different temperatures (42, 50, and 56°C) for 10 min.

All samples for stress test were serially diluted and spread on TSA plates.

### 2.8 Antimicrobial susceptibility test

The minimum inhibitory concentration (MIC) was determined in 96-well plates using commercial kit (Biofosun Diagnostics) as protocol. The freshly grown bacteria on TSA plates were picked into sterile normal saline, and calibrated to OD_600_ = 0.1 in nutrient broth medium (containing serum and NAD). Then 100 μL of the calibrated bacteria suspension was added to each well, and incubated in a constant temperature incubator at 37°C for 48 h. A total of 16 antibacterial drugs are used: Ampicillin (0.25–512 μg/mL); amoxicillin/clavulanate (0.25/0.12–512/256 μg/mL); gentamicin (0.25–512 μg/mL); spectinomycin (0.25–512 μg/mL); tetracycline (0.25–512 μg/mL); florfenicol (0.25–512 μg/mL); Sulfafurazole (0.25–512 μg/mL); trimethoprim/sulfamethoxazole (0.06/1.2–32/608 μg/mL); Ceftiofur (0.12–256 μg/mL); ceftazidime (0.12–256 μg/mL); enrofloxacin (0.015–32 μg/mL); ofloxacin (0.03–64 μg/mL); meropenem (0.008–16 μg/mL); Apramycin (0.06–128 μg/mL), Colistin (0.12–256 μg/mL); acemequine (1–512 μg/mL).

### 2.9 Biofilm assay

The strains Δ*L32* and ZJ1208 were inoculated in 5 mL of TSB medium, and cultured overnight at 37°C, 200 rpm. The cell density was adjusted to OD_600_ = 0.65 (ΔL32 2.1 × 10^9^ CFU/mL, ZJ1208 2.7 × 10^9^ CFU/mL). Subsequently, 100 μL of the suspension was dispensed into a 96-well plate (Corning, 3,599) with 8 wells allocated for each strain. A negative control group with no bacteria in the medium was also included. The plate was then incubated at 37°C for 48 h. Following incubation, the medium was removed, and each well underwent two gentle washes with ultrapure water (200 μL per well). After drying, 100 μL of 1% crystal violet solution was added to each well and allowed to stain for 2 min before being washed three times with ultrapure water. Finally, 100 μL ethanol (70%, v/v) was added to each well, and the optical density at wavelength of 590 nm (OD_590_) was measured using a microplate reader (SpectraMax M5, Molecular Devices).

### 2.10 Virulence assays and animal ethics statements

The experimental animals used in this study were 4-week-old male BALB/c mice, and were obtained from Shanghai Slake Laboratory Animal Co, Ltd. The mice were randomly assigned into three groups of 10 each, and intraperitoneally injected with *G. parasuis* (3.2 × 10^9^ CFU/mL) resuspended in 0.5 mL saline; the control group received saline injection only. Survival rates were monitored every 12 h throughout the experiment period. All mice were provided with *ad libitum* access to food and water, and humane practices were employed to minimize their suffering. At the end of the experiment, all mice were euthanized. This study was approved by the Animal Ethics Committee of Zhejiang Academy of Agricultural Sciences (Approval Number: 2023ZAASLA86).

### 2.11 Statistical analysis

Statistical analysis was conducted using GraphPad Prism software (one-way ANOVA; Tukey’s *post hoc* test, ***p < 0.001, **p < 0.01, *p < 0.05). The results were presented as mean ± standard deviation (SD).

## 3 Results

### 3.1 Construction of *L32* gene deletion mutant

**Figure 2:**
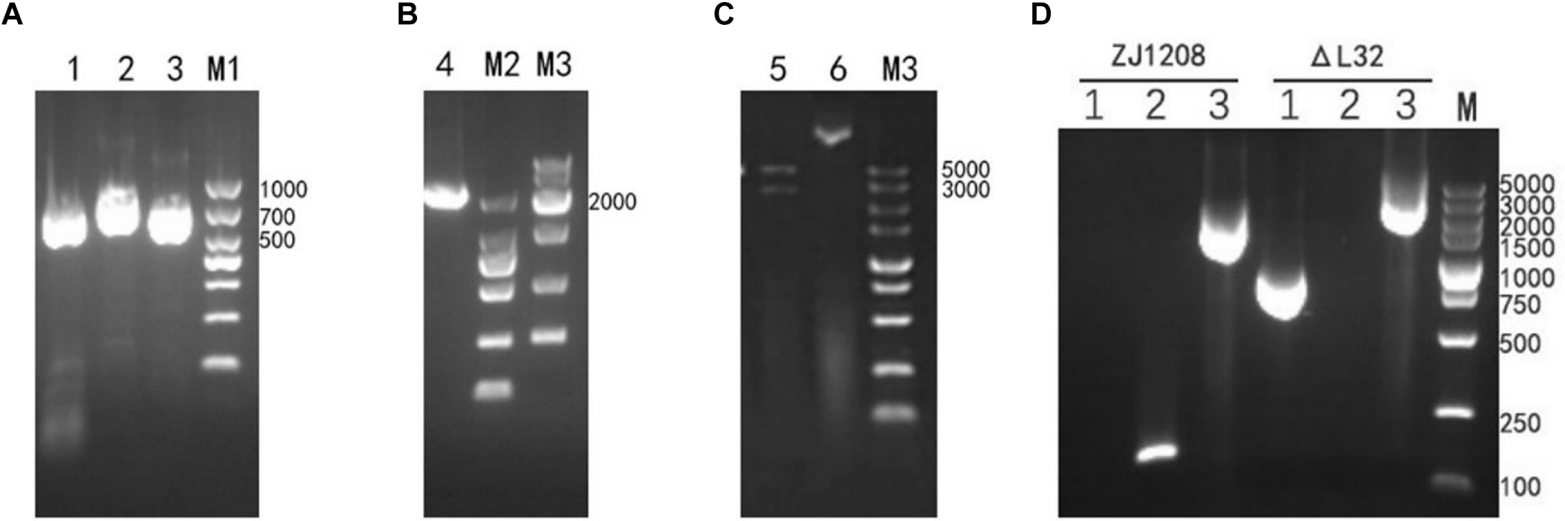
Construction and Identification of Δ*L32* mutant. **(A)** Upper and lower homologous arms were amplified from the genome DNA of ZJ1208, and the KanR cassette fragment was amplified from the pET-28a plasmid. Lane 1: amplicon of upstream homologous arm (729 bp), lane 2: amplicon of KanR cassette (848 bp), lane3: amplicon of downstream homologous arm (721 bp). **(B)** The amplicon of overlap PCR for ligation of *L32*-UP+KanR+*L32*-Down. **(C)** The recombinant plasmid pEASY-*L32* was identified through restriction enzyme digestion with *BamH* I/*Xho* I (Lane 5), while undigested plasmid served as a control (Lane 6). **(D)** PCR identification of the replacement of *L32* by KanR cassette. Lane 1: amplicon of KanR cassette (848 bp); lane 2: amplicon of *L32* gene (162 bp); lane 3: an increase in PCR product size from 1,612 to 2,298 bp after KanR cassette substitution of the *L32* gene, as detected by *L32*-UF and *L32*-DR primers.

The upstream and downstream homology arms and KanR cassette fragments were obtained through amplification (Figure 2A). Subsequently, the upstream and downstream homologous arms along with the KanR cassette fragment were fused via overlapping PCR to generate a fragment of 2,298 bp (Figure 2B). Then recombinant plasmids were validated by restriction endonuclease digestion (Figure 2C), and the accuracy of the inserted sequence was further validated by sequencing (data not shown). Following natural transformation, the colonies cultivated on kanamycin supplemented plates were selected to ascertain the knockout of the *L32* gene and insertion of the KanR cassette (Figure 2D). Colony present with KanR cassette and absent with *L32* gene was selected, and was designated as Δ*L32*.

### 3.2 Polar effect determination in Δ*L32*

**Figure 3:**
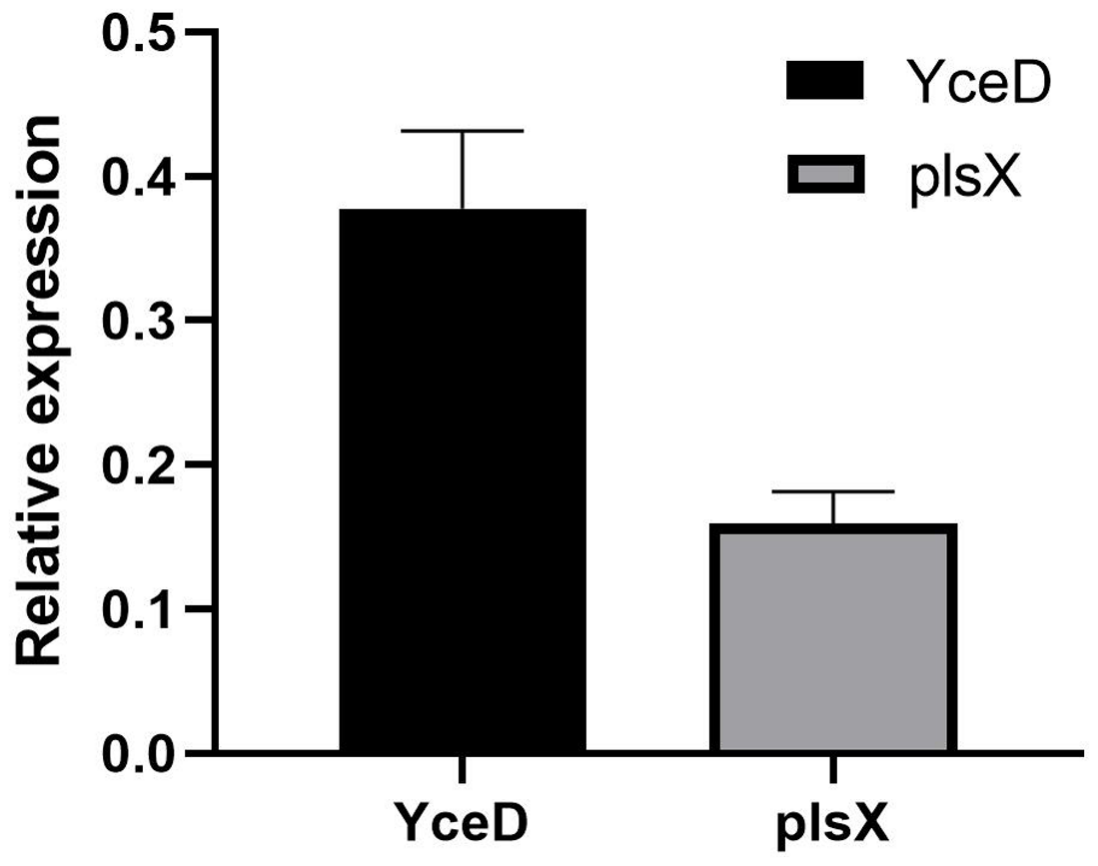
Relative expression levels of upstream (*YceD*) and downstream genes (*plsX*) of *L32* gene. Samples were normalized to the 16S rRNA gene as a control. Relative expression level is represented as 2^−(∆∆ CT)^, and the mean ± standard deviation was indicated for three independent experiments.

The relative expression levels of the upstream gene *YceD* and downstream gene *plsX* of the *L32* gene is represented by 2^−(∆∆ CT)^. The results showed that *L32* gene deletion did not cause significant differences in gene expression levels of both upstream and downstream genes (Figure 3). Hence, deletion of *L32* did not cause polar effect.

### 3.3 Comparison of growth

**Figure 4:**
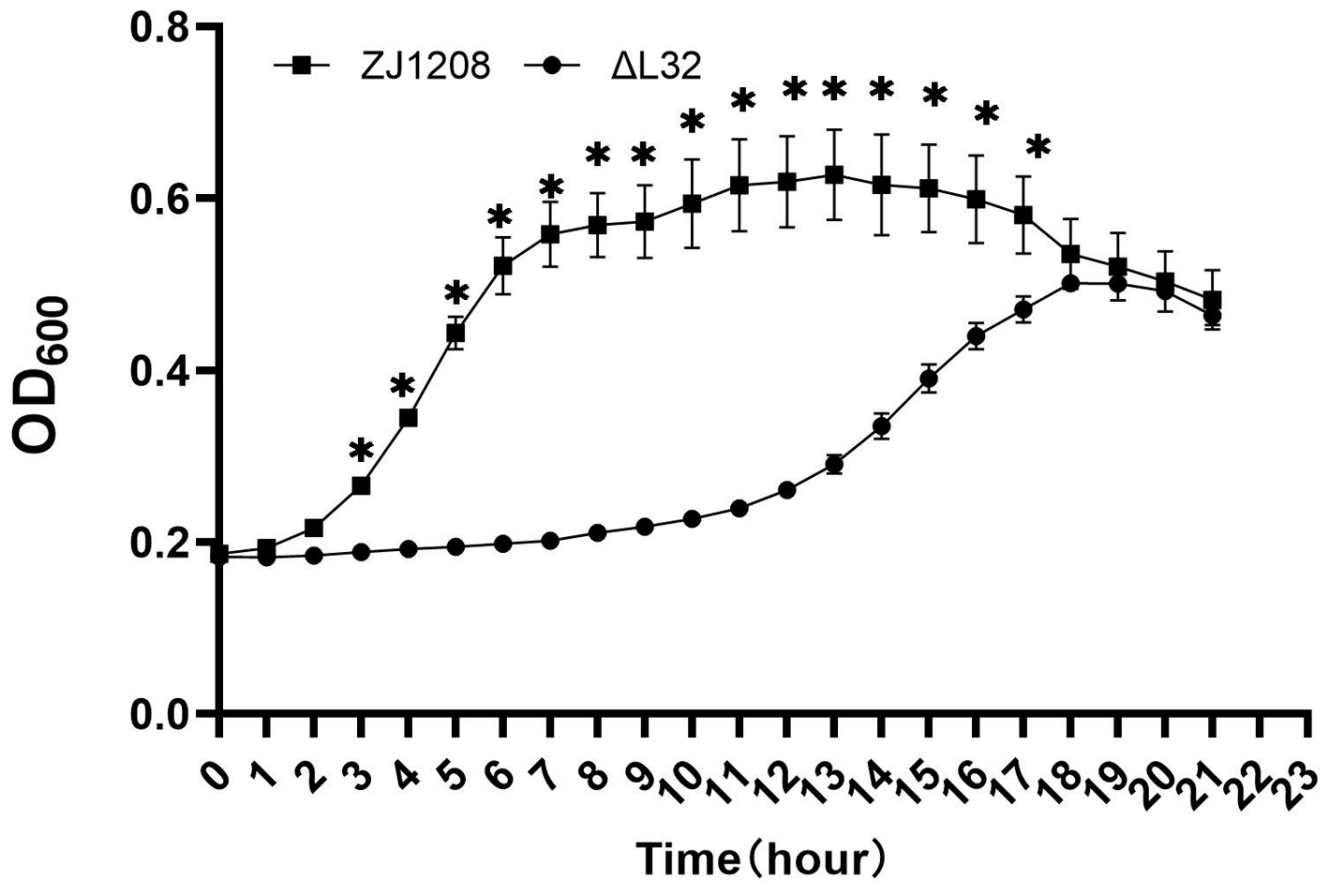
Growth curves of Δ*L32* and ZJ1208. The cultures were inoculated in fresh TSB medium at a ratio of 1:50 in triplicates, and cultured in a microbial growth analyzer at 37°C, and OD_600_ value was measured at 1 h interval, and the mean ± standard deviation was indicated (*p < 0.05).

The growth curve of Δ*L32* is clearly different compared with that of ZJ1208, and the lag phase of Δ*L32* was extended by about 8 h (Figure 4). ZJ1208 achieves its stationary phase with OD_600_ value of 0.65 after 7 h of culture, whereas Δ*L32* reaches its stationary phase with OD_600_ value of 0.5 after 18 h.

### 3.4 Colony morphology

**Figure 5:**
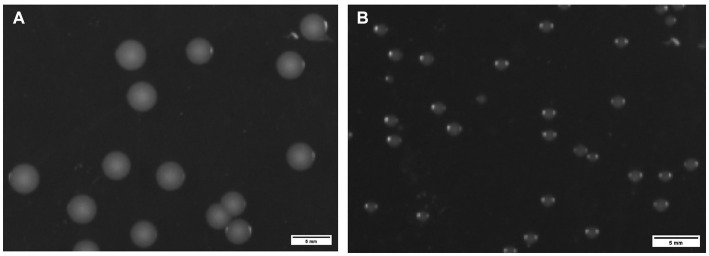
Colony morphology of ZJ1208 wild strains **(A)** and Δ*L32* **(B)**. *G. parasuis* was streaked on TSA plates and incubated at 37°C for 48 h. Colonies were observed by a chemiluminescence imaging system, and ImageJ was used to process images.

Translucent, smooth and rounded small colonies (approximately 3 mm in diameter) were seen after 48 h at 37°C for ZJ1208 (Figure 5A), while it took 48 h for Δ*L32* to reach a diameter of about 1 mm (Figure 5B).

### 3.5 Transmission electron microscopy

**Figure 6:**
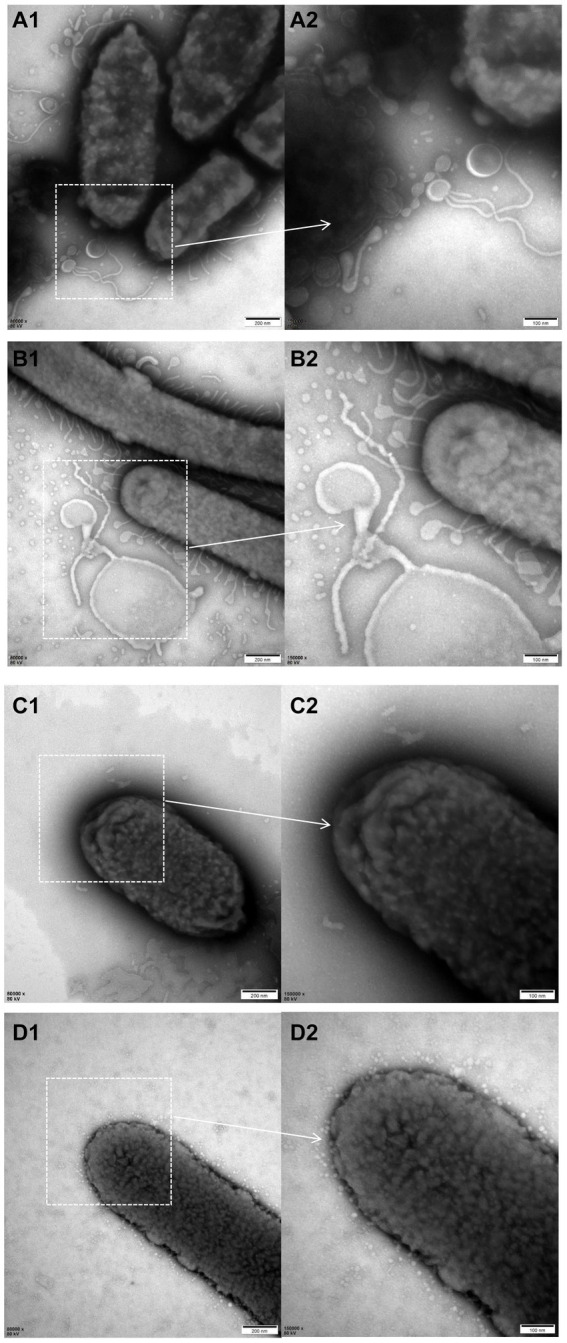
TEM photographs of *G. parasuis* cells of Δ*L32* **(A,B)** and ZJ1208 **(C,D)** from solid **(A,C)** and liquid medium **(B,D)**. Cells were stained with 2% phosphotungstic acid for 8 s before observation by TEM (magnification, 80,000× for the left column and 150,000× for the right column).

The electron microscopy results revealed a higher abundance of outer membrane vesicles (OMVs) when cultured with liquid medium compared to TSA plates either for ZJ1208 or Δ*L32*. Interestingly, regardless of the growth condition, Δ*L32* secreted more OMVs which showed an increased pleomorphism, including bead-string shaped, mallet-shaped and long tubular shape etc. (Figure 6). In contrast, ZJ1208 produced fewer and smaller OMVs with uniform shape and size, mostly appearing as regular near-round structures.

### 3.6 Osmotic pressure, oxidation tolerance and heat shock experiments

**Figure 7:**
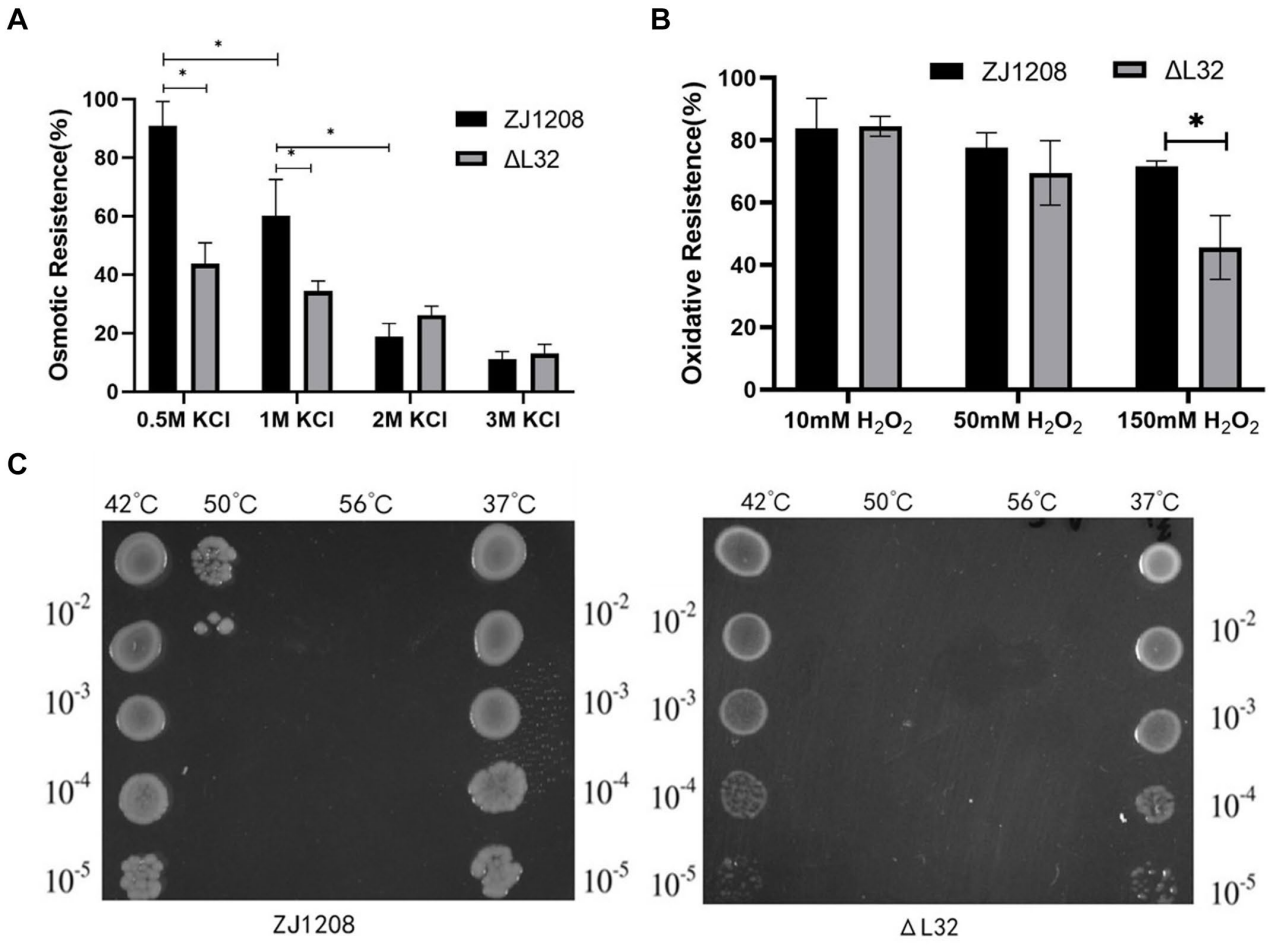
Stress susceptibility of Δ*L32* and ZJ1208. The *G. parasuis* culture were adjust to the OD_600_ value of 0.65 in TSB medium. **(A)** Bacteria culture was resuspended in equal volume of TSB containing different concentration of KCl, and incubated at 37°C for 2 h; **(B)** bacteria culture was resuspended in equal volume of TSB containing different concentration of H_2_O_2_, and incubated at 37°C for 1 h; **(C)** 1 mL of bacterial culture was incubated at different temperatures (42, 50, and 56°C) for 10 min, 37°C as a control group. All cultures above for stress test were serially diluted and spread on TSA plates. All the above measurements were conducted three times. The bar chart represents the mean ± standard deviation of three independent experiments. Statistical analysis was performed with multiple *t*-tests, *p < 0.05.

The results showed that the Δ*L32* was significantly more susceptible to osmotic pressure under the condition of 0.5 M and 1 M KCl, and the resistance to osmotic pressure of ZJ1208 was decreased dramatically when KCl concentration was increased to 2 M (Figure 7A). There is no significant difference in oxidation tolerance under low concentration of H_2_O_2_, while Δ*L32* was significantly more susceptible to 150 mM H_2_O_2_ compared with ZJ1208 (Figure 7B). Similarly, *L32* was more susceptible to high temperature of 50°C, while both strains could not survive when the temperature raised to 56°C (Figure 7C). This indicates that the absence of *L32* gene affects the stress tolerance of *G. parasuis*.

### 3.7 Antimicrobial susceptibility test

The minimum inhibitory concentration (MIC) of Δ*L32* decreased in respect to all aminoglycoside antibiotics used in this study, including spectinomycin, Apramycin, gentamycin. Higher susceptibility was also observed for Δ*L32* to sulfonamide antibiotics (sulfafurazole) in this study (Table 3). The mutant shown no difference in resistance to other antibiotics used in this study compared to ZJ1208. This suggests that *L32* plays an important role in resistance to these two types of antibiotics.

**Table 3:** MIC of antimicrobials.

Antimicrobials	MIC (μg/mL)	
	ZJ1208	Δ*L32*
Ampicillin	0.25	0.25
Amoxicillin/clavulanate	0.25/0.12	0.25/0.12
Gentamicin	1	0.5
Tetracycline	1	1
Spectinomycin	1(S)	0.25* (S)
Florfenicol	0.25	0.25
Sulfafurazole	16 (S)	4* (S)
Trimethoprim/sulfamethoxazole	1/19	0.5/9.5
Ceftiofur	0.12	0.12
Ceftazidime	0.12	0.12
Enrofloxacin	2	1
Ofloxacin	2	2
Meropenem	0.008	0.008
Apramycin	16	2*
Colistin	0.12	0.12
Acemequine	1	1

The experiment was repeated three times, and statistical analysis was performed with *t*-tests *P < 0.05. MIC breakpoints referred to literature (15). S, susceptible; R, resistant.

### 3.8 Virulence assays

**Figure 8:**
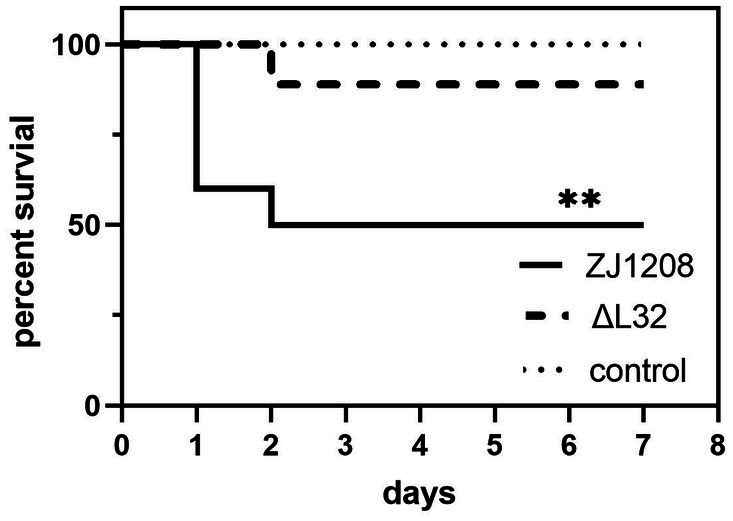
Survival curves of mice challenged with ZJ1208 and Δ*L32*. Mice were intraperitoneally injected with *G. parasuis* (3.2 × 10^9^ CFU/mL) resuspended in 0.5 mL saline; the control group received saline injection only. The number of survivals is recorded every 12 h for 7 days post challenge. The difference in survival rates between *L32* and ZJ1208 is analyzed by GraphPad Prism 9 (**p < 0.01), using log-rank test.

On the first day of challenge, four mice succumbed to infection in the ZJ1208 group, followed by an additional death on the second day. In contrast, only one mouse died on the second day in the Δ*L32* group. The Δ*L32* exhibited a significant reduction of mortality by 40% compared to that of ZJ1208 (Figure 8). No mortality was observed in the negative control group (normal saline).

## 4 Discussion

*G. parasuis* mainly infects piglets before and after weaning, as well as during the nursery stage, and is one of the main cause of death in piglets aged 2 weeks to 4 months. It has become one of the main bacterial diseases that harm the pig farming industry, causing serious economic losses to the global pig farming industry (16). At present, the pathogenic mechanism of this bacterium is not fully understood, and the cross-protection effect of the vaccine against heterologous strains is poor (17). Therefore, the elucidation of the pathogenic mechanism of this bacterium will help to discover drug targets and develop more effective new vaccines.

Ribosomal proteins are important components of ribosomes and play key roles in ribosome biogenesis and protein translation. It has been reported that ribosomal proteins are related to the growth rate of bacteria, and results of the effect of L32 on growth rate are inconsistent (6–8). So we wanted to know whether L32 also affects the growth rate of *G. parasuis*. In this study, the growth curve of Δ*L32* is clearly different compared with that of ZJ1208, which indicates that the *L32* gene is important for the growth and reproduction of this bacterium.

Disturbances in the intracellular environment will lead to an increase in outer membrane vesicles to expel unwanted substances. Misfolded protein products may be selectively enriched in vesicles. By increasing the amounts of vesicles, toxic substances are removed, thereby protecting the bacteria (18, 19). Changes in cell membrane structure and composition can also cause increased outer membrane vesicles production, such as *DegP* deletion (18), *ampG* and *amiD* gene mutations (20), and *rfaC* and *rfaG* gene mutations (21). The production of outer membrane vesicles is considered to be a result of bacterial emergency response (22). Ribosomal protein L32 of *Saccharomyces cerevisiae* binds and regulates the splicing and translation of its own gene transcripts (7). After knocking out the *L32* gene in non-sexually flocculating fission yeast cells, the cell wall became thicker and the composition of the cell wall increased (23). There is currently limited research on whether the absence of ribosomal proteins leads to an increase in vesicles. However, in yeast research, the absence of L32 can lead to changes in cell wall composition, so we speculate that ZJ1208 may also experience a similar situation after losing L32, indirectly leading to an increase in outer membrane vesicles.

The *rpmF* gene encoding ribosomal protein L32 in both *Escherichia coli* and *Rhodobacter capsulatus* is located in the same operon with the *plsX* gene encoding a protein involved in membrane lipid synthesis, and it is speculated that there is synergy between ribosome and cell membrane (24, 25). Therefore, we speculate that the cell membrane function of Δ*L32* may be affected, and we found that the resistance to osmotic pressure and oxidative tolerance of Δ*L32* was decreased, which indicated that the function of cell membrane was impaired. The susceptibility of Δ*L32* to heat shock was increased, potentially attributed to the impact of *L32* deletion on the expression of heat shock proteins, resulting in compromised thermotolerance.

Macrolides have been shown to interact with many proteins in the 70S ribosome, including L32 (10). In *Streptococcus pneumoniae*, the L32 protein has been shown to contribute to the SOL (solithromycin) resistance phenotype, and mutations in L32 lead to increased resistance to SOL (9). In the experiment, we found that the sensitivity of L32 to aminoglycoside and sulfonamide antibiotics changed. There is little research on the relationship between ribosomal protein L32 and aminoglycosides and sulfonamides, and its mechanism still needs to be elucidated.

Biofilm formation of *H. parasuis* field strains varies extensively, and more than 30% of the strains could not form biofilm. Meanwhile, 66.4–69.2% of the tested strains could form biofilm, most of which performed weak biofilm-forming ability (26, 27). The research of the well-characterized virulent and non-virulent strains of *H. parasuis* shows that non-virulent strains formed significantly more biofilms than virulent strains (28). Certain gene deletion can alter the biofilm formation property of *H. parasuis* by either increase or decrease the biofilm formation (29–31). The ZJ1208 used in this study is isolated from the lung of a diseased pig and belongs to serovar 13 which has been traditionally proposed as a highly virulent serovar (1). The affect of *L32* deletion on biofilm formation was determined in this study, and deletion of *L32* did not alter the poor biofilm formation property of ZJ1208 (Supplementary Data Sheet S1).

Although gene sequencing (Supplementary Data Sheet S2) was performed to ensure the accuracy in the plasmid construction procedure, there is still possibility that other unwanted mutation take place during the following process, and unwanted mutations may also lead to phenotypic changes. Hence, whole genome sequencing of the strains was conducted to exclude this possibility. No strain specific gene was observed after comparation of whole genome sequence of ZJ1208 (Supplementary Data Sheet S3) and ΔL32 (Supplementary Data Sheet S4). Meanwhile, one deletion mutation in the tandem repeat sequence of a virulence associated trimeric autotransporter and several SNP in other genes or non-coding sequence were found in ΔL32 (Supplementary Data Sheet S5), which is not correlated to the phenotype variation in this study. Limitation of this study is that only one strain was used, and the results may not applicable to other strains. Hence, the function of *L32* in *G. parasuis* is still to be fully characterized.

To our knowledge, there is no article showing a clear connection between the *L32* gene and bacterial virulence. This study found that its pathogenicity to mice was reduced, indicating that *L32* is a virulence-associated factor which contributes to bacterial fitness in host environments. In summary, we found that *L32* is associated with growth, stress and antibiotic resistance, and virulence in *G. parasuis*.

## Data Availability

The original contributions presented in the study are included in the article/Supplementary material, further inquiries can be directed to the corresponding authors.
