# Supplementary material for: Ribosomal protein L32 contributes to the growth, antibiotic resistance and virulence of Glaesserella parasuis
Source: Front Vet Sci. 2024 Aug 26;11:1361023. doi: 10.3389/fvets.2024.1361023 (PMC11381497; doi:10.3389/fvets.2024.1361023)
Supplement: Supplementary file 1 [file Data_Sheet_1.docx]

**Biofilm experiments**

The crystal violet staining of biofilm in the 96-well plate shown that little biofilm was formed for both ZJ1208 and Δ*L32* (Fig. 7A). The results showed that the biofilm production of Δ*L32* is similar to that of ZJ1208 (Fig. 7B). There is no significant difference between ZJ1208 and Δ*L32* in terms of biofilm production.


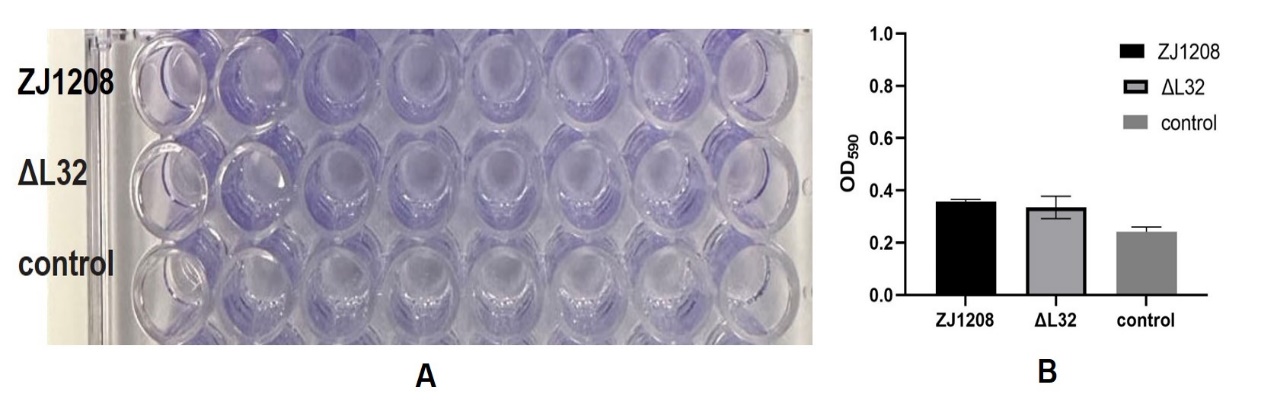


Fig. 7 Biofilm formation of ZJ1208 and Δ*L32*. (A)Crystal violet staining; (B) Biofilm formation under static conditions in micro titer plates for G.*parasuis* were stained with crystal violet, and quantified by measure the OD_590_ after dissolving in ethanol. All the above measurements were conducted three times. The bar chart represents the mean ± standard deviation of three independent experiments.
