# Supplementary material for: Ribosomal protein L32 contributes to the growth, antibiotic resistance and virulence of Glaesserella parasuis
Source: Front Vet Sci. 2024 Aug 26;11:1361023. doi: 10.3389/fvets.2024.1361023 (PMC11381497; doi:10.3389/fvets.2024.1361023)
Supplement: Supplementary file 2 [file Data_Sheet_2.docx]

Sequencing results of the recombinant plasmid used for transformation of ZJ1208

ACGGCGAACGTTTTAACATATAAGCGGTGGGATTGCTACTAAATTTCACAAAATAATGTAATAGATACATATAAGCGGCATGATTCTACGGATTTTTTACAAATCCAGATTTTCTCTTTGACTAGTTACTTCAAAATCTGTAATATTCGCACCCTATGCAAAAGGTAAAACTACCCCTAACTATTGACCCATATAAAGACGCTCAGCGTCGAATGGACTACGAAGGCTACATTTCTGCGAACCTACTTAGCCGTTTGGGTGAATCTGTTGGTCAAATGCTGGGCGATGCACAAGTTACTTTATCGTTATATGTTGATCCGCAAAAACTCACAGTGGTTAAAGGCACTGCGAAAGTTGATGTGGAGCTAGACTGTCAACGTTGTAGTAACCCATTTACGCAAACACTCGACTGCACATTTTGTTTCAGTCCAGTGTCTAATATGGATCAGGCGGACAATTTGCCCGAGATTTATGAACCAATTGAAGTTGATTCTTTTGGTGAGATAAATTTGCTAGATATGATAGAAGACGAATTCATTCTGTCATTACCCCTAGTGCCGATGCATACCGATGAACACTGTGAAGTGTCCGAAAGCGAATTAGTTTTTGGCGAATTACCAGAAGAACTGGCAAAGAAACCAAATCCTTTCGCAATATTAGCTAATTTAAAGAAAAACTAGATCTAGTAGGAGTATAGCCAatgagccatattcaacgggaaacgtcttgctctaggccgcgattaaattccaacatggatgctgatttatatgggtataaatgggctcgcgataatgtcgggcaatcaggtgcgacaatctatcgattgtatgggaagcccgatgcgccagagttgtttctgaaacatggcaaaggtagcgttgccaatgatgttacagatgagatggtcagactaaactggctgacggaatttatgcctcttccgaccatcaagcattttatccgtactcctgatgatgcatggttactcaccactgcgatccccgggaaaacagcattccaggtattagaagaatatcctgattcaggtgaaaatattgttgatgcgctggcagtgttcctgcgccggttgcattcgattcctgtttgtaattgtccttttaacagcgatcgcgtatttcgtctcgctcaggcgcaatcacgaatgaataacggtttggttgatgcgagtgattttgatgacgagcgtaatggctggcctgttgaacaagtctggaaagaaatgcataaacttttgccattctcaccggattcagtcgtcactcatggtgatttctcacttgataaccttatttttgacgaggggaaattaataggttgtattgatgttggacgagtcggaatcgcagaccgataccaggatcttgccatcctatggaactgcctcggtgagttttctccttcattacagaaacggctttttcaaaaatatggtattgataatcctgatatgaataaattgcagtttcatttgatgctcgatgagtttttctaaTCAATTGGATCTCGTAGAGGTTTAAATTGACTCATCTAACTCTCGCGTTAGATGCGATGGGCGGGGACTTTGGTCCCCGTATTACTATCCCTGCATTATCACTTGCGTTGGCTCAACATCCAATACTCAGTTGCATCTTGTTCGGCGACCAAGCTGAAATATCCCCCTATCTCAATAAACTTTCTCCCGATATTCAACAACGTATCGAATTAGTCCATACAACAAAAGTGATCGAAGCAGACTTGCCTTTTGTGCAAGCGATTCGTCAAAGTAAAGGCAGTTCAATGCGTTTAGCGATTGAAGCGGTAGAAAATGGAAATGCTCAAGGTTGTGTAAGTGGCGGAAATACAGGTGTACTGATGGGGCTAGCAAAACAATTGATTGAACCCTTGCCAAACATTGATCGTCCCGCATTAACATCTTTGATTCCAACAATTAACGGCAACTCAAGTGTGATGTTAGATCTTGGTGCAAACGTTGAAGCAGATAGTGAACTATTGCTTCAGTTTGCGGAAATGGGCAATGTGTTTGCAGAAGTGATGTTGGATTTGGTCTATCCACGTTTAGCGTTGCTGAATATTGGAACAGAAGAGCATAAAGGAACACAGCAAATTCGAGATGCTCACCAGCAATTAAAACAATGTAATCATCTAAATTACATTGGCTTCTTAGAAGGGGATAAATTAATGAATCATCTTGC
