# Supplementary material for: Ribosomal protein L32 contributes to the growth, antibiotic resistance and virulence of Glaesserella parasuis
Source: Front Vet Sci. 2024 Aug 26;11:1361023. doi: 10.3389/fvets.2024.1361023 (PMC11381497; doi:10.3389/fvets.2024.1361023)
Supplement: Supplementary file 3 [file Data_Sheet_3.docx]

whole genome sequencing on the WT and L32 deletion strain was conducted. We found through gene sequence alignment that compared with the wild-type strain ZJ1208, except for the replacement of the L32 gene by KanaR, an additional T was inserted at the 1694929 position (Fig. 1) and a T was missing at the 926250 base position (Fig. 2) in the L32 deletion strain genome sequence. The gene encoding trimeric autotransporter adhesin located at position 447649 has undergone varying degrees of deletion and insertion (Fig.3), but it is not related to the phenotype involved in our study. Therefore, we can infer that the changes in phenotypes are not caused by the mutation in other genes. To further confirm, it may be more appropriate to measure the transcription level of the genome.


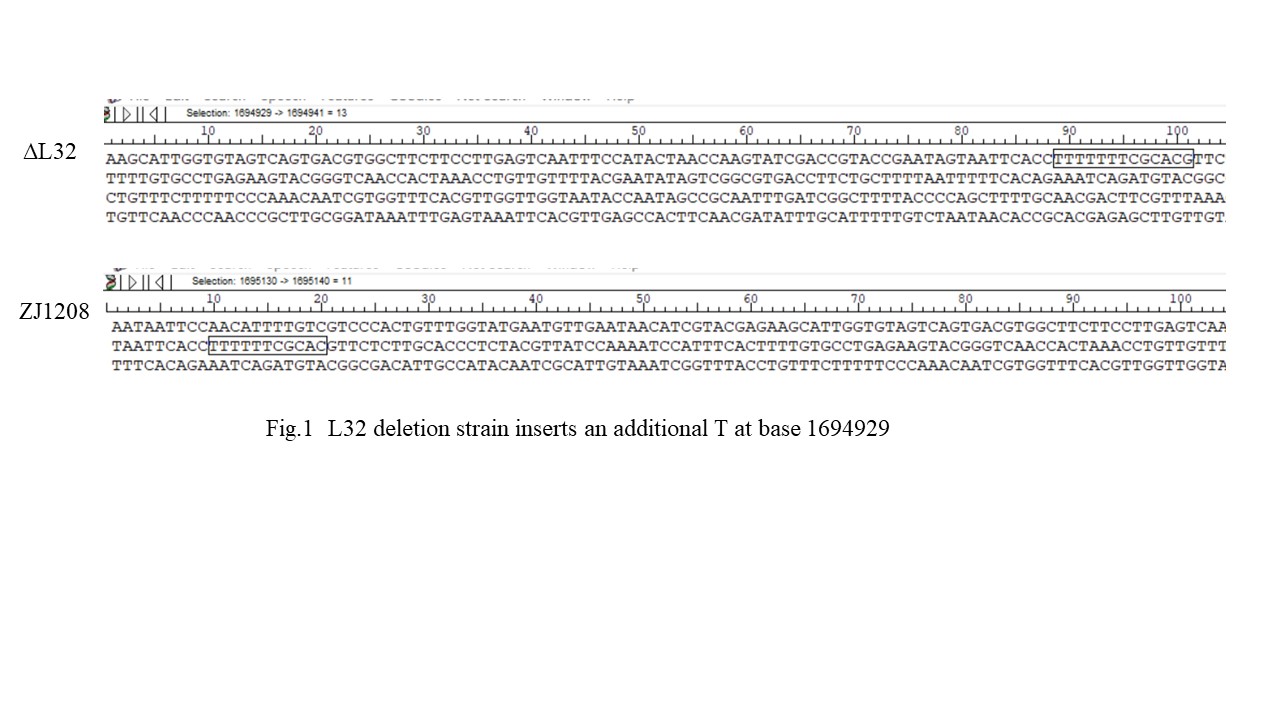

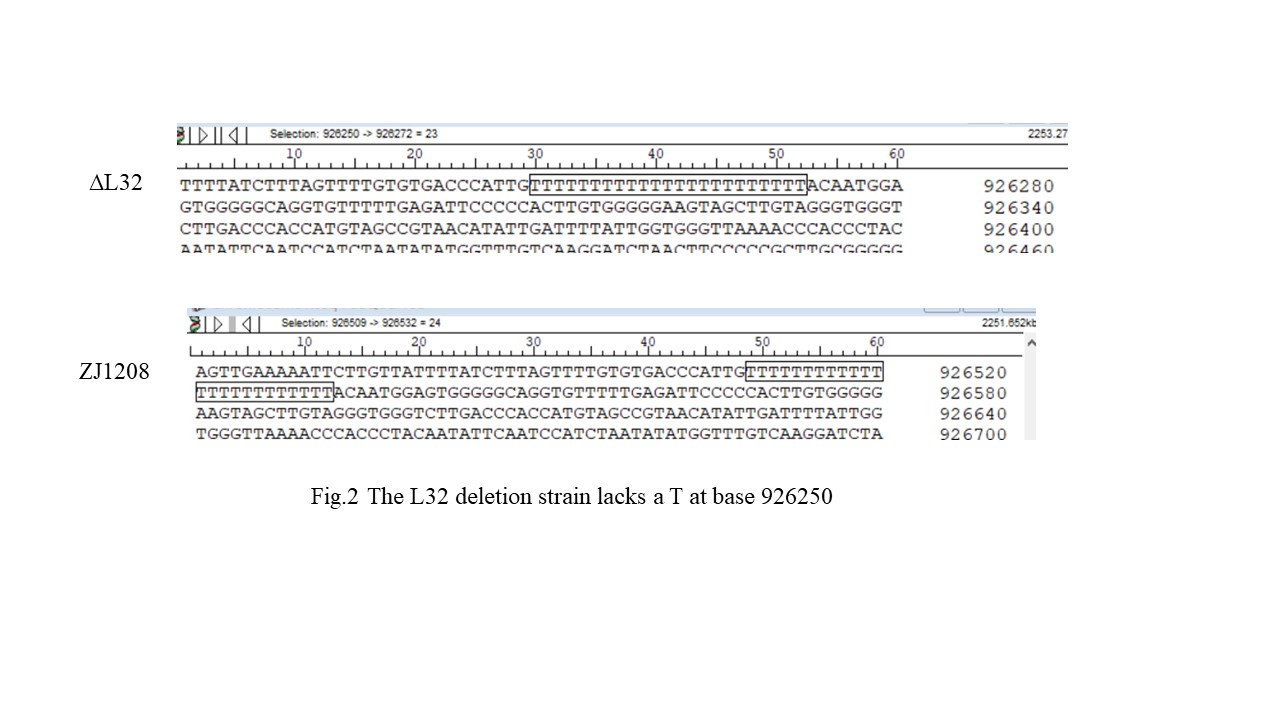


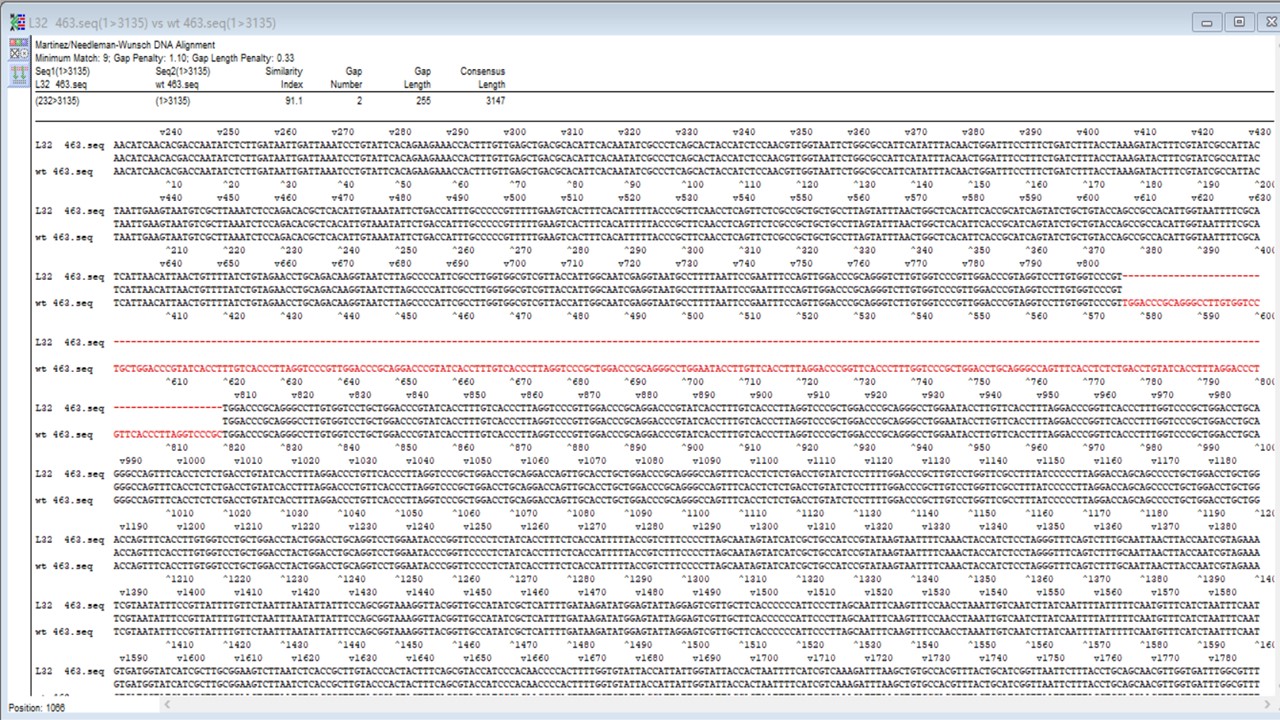


Fig.3 Deletion mutation in a trimeric autotransporter
